# Determining the Distribution of Fluorescent Organic Matter in the Indian Ocean Using *in situ* Fluorometry

**DOI:** 10.3389/fmicb.2020.589262

**Published:** 2020-12-23

**Authors:** Masahito Shigemitsu, Hiroshi Uchida, Taichi Yokokawa, K. Arulananthan, Akihiko Murata

**Affiliations:** ^1^Physical and Chemical Oceanography Research Group, Global Ocean Observation Research Center, Research Institute for Global Change, Japan Agency for Marine-Earth Science and Technology, Yokosuka, Japan; ^2^Super-cutting-edge Grand and Advanced Research Program, Institute for Extra-cutting-edge Science and Technology Avant-garde Research, Japan Agency for Marine-Earth Science and Technology, Yokosuka, Japan; ^3^National Institute of Oceanography and Marine Sciences, National Aquatic Resources Research and Development Agency, Colombo, Sri Lanka

**Keywords:** FOM, *in situ* fluorometer, Indian Ocean, water mass analysis, microbial respiration

## Abstract

In order to determine the dynamics of marine fluorescent organic matter (FOM) using high-resolution spatial data, *in situ* fluorometers have been used in the open ocean. In this study, we measured FOM during the Global Ocean Ship-based Hydrographic Investigations Program (GO-SHIP) expedition from early December 2019 to early February 2020, using an *in situ* fluorometer at 148 stations along the two meridional transects (at ∼80 and ∼57°E) in the Indian Ocean, covering latitudinal ranges from ∼6°N to ∼20°S and ∼30 to ∼65°S, respectively. The FOM data obtained from the fluorometer were corrected for known temperature dependence and calibrated using FOM data measured onboard by a benchtop fluorometer. Using the relative water mass proportions estimated from water mass analyses, we determined the intrinsic values of FOM and apparent oxygen utilization (AOU) for each of the 12 water masses observed. We then estimated the basin-scale relationship between the intrinsic FOM and the AOU, as well as the turnover time for FOM in the Indian Ocean (410 ± 19 years) in combination with the microbial respiration rate in the dark ocean (>200 m). Consistent to previous estimates in the global tropical and subtropical ocean, the FOM turnover time obtained is of the same order of magnitude as the circulation age of the Indian Ocean, indicating that the FOM is refractory and is a sink for reduced carbon in the dark ocean. A decoupling of FOM and AOU from the basin-scale relationship was also observed in the abyssal waters of the northern Indian Ocean. The local variability may be explained by the effect of sinking organic matter altered by denitrification through the oxygen-deficient zone on enhanced abyssal FOM production relative to oxygen consumption.

## Introduction

The carbon inventory of marine dissolved organic matter (DOM) is estimated to be approximately 662 Pg C, which is the second largest among the bioreactive carbon reservoirs in the ocean (Hansell et al., 2009). Most DOM exists as refractory DOM (RDOM) (Hansell et al., 2012), which is thought to be produced by microbial mineralization of organic matter that is produced in the sunlit surface ocean and accumulates in deep/abyssal waters in the course of meridional overturning circulation (Hansell et al., 2009, 2012). As a result, RDOM plays an important role in sequestering atmospheric CO_2_ in deep/abyssal waters via the mechanism called the “microbial carbon pump (MCP)” (Jiao et al., 2010). Thus, a better understanding of the behavior of marine RDOM is needed for deepening our knowledge of the MCP.

Some constituents of RDOM can be detected as chromophoric DOM (CDOM) or fluorescent DOM (FDOM). CDOM is a fraction of DOM that absorbs ultraviolet–visible light, while FDOM is a fraction of CDOM that fluoresces. RDOM in the ocean has a lifetime of hundreds to thousands of years (e.g., Hansell et al., 2012), indicating that large-scale observations are indispensable for tracing basin or global scale RDOM behavior and for determining the synoptic dynamics of RDOM. To date, several large-scale observations regarding FDOM/CDOM have been conducted. Yamashita and Tanoue (2008) determined that marine humic-like FDOM (measured at 320 nm excitation and 420 nm emission) correlated linearly with apparent oxygen utilization (AOU) along a transect in the Pacific covering a latitudinal range from ∼60°N to ∼55°S. Nelson et al. (2010) showed that CDOM (measured as an absorption coefficient at 325 nm) was also positively correlated with AOU in the Indian and Pacific Oceans, but not in the Atlantic Ocean. Jørgensen et al. (2011) clarified that humic-like fluorophores had positive correlations with AOU by using the global distributions of FDOM determined with excitation-emission matrix (EEM) fluorescence and parallel factor analyses (PARAFAC). Catalá et al. (2015a, b) also showed the global tropical and subtropical distributions of EEM measurements treated by PARAFAC analyses and CDOM (same as in Nelson et al., 2010). The first three studies demonstrated that FDOM/CDOM are produced in the ocean interior via microbial respiration, based on positive correlations between FDOM and AOU or between CDOM and AOU. These studies analyzed FDOM/CDOM and AOU data on a basin or global scale by combining them, but without considering the differences in the specific values of each parameter between the water masses. This makes it difficult to obtain a characteristic basin scale or global scale relationship between FDOM and AOU, or between CDOM and AOU. The difficulty lies in the fact that the analytical method used does not consider the mixing of several water masses in a given location, and the possibility that intermediate and deep/abyssal water masses are transported to a given basin from other ocean basins. On the other hand, the latter two studies determined the characteristic values of FDOM/CDOM and AOU for each water mass encountered, obtaining the global relationships between FDOM and AOU and between CDOM and AOU. However, although the methods used by Catalá et al. (2015a, b) seem reasonable, the geographical and vertical resolutions of their FDOM/CDOM data were not very high and the data coverage was restricted to the subtropical and tropical regions. Especially in the Indian Ocean, their transect survey was conducted only in the subtropical region, which indicates that the basin-scale information on FDOM/CDOM is insufficient.

In order to increase the spatial resolution of marine FDOM/CDOM data both geographically and vertically, *in situ* fluorometry is a promising technique. Recently, *in situ* fluorometers have been used in open ocean studies coupled with CTD systems (Yamashita et al., 2015; Nelson and Gauglitz, 2016) and autonomous profiling Bio-Argo floats (Xing et al., 2012). Yamashita et al. (2015) measured the vertical distributions of FDOM at several stations in the northwestern Pacific and showed the importance of correcting the temperature dependence of an *in situ* fluorometer, based on the method proposed by Watras et al. (2011). Xing et al. (2012) analyzed data from the upper 400 m of the Pacific, Atlantic, and Mediterranean to examine the potential for obtaining the CDOM absorption coefficient from *in situ* fluorometer-derived CDOM data. Although Nelson and Gauglitz (2016) collected vertical FDOM data on a large scale, they only compared the data with the manually measured CDOM absorption coefficient at 325 nm. Therefore, studies that describe vertical FDOM data, obtained by *in situ* fluorometry on a large scale, to understand its behavior in the ocean interior are currently lacking.

In this context, this study focuses on making large-scale observations and collecting FDOM data at high spatial resolutions using an *in situ* fluorometer in the Indian Ocean, where the basin-scale information on FDOM is insufficient. This study aims to: (1) establish *in situ* fluorometer application in the open ocean, (2) clarify the characteristic FDOM values for each of the water masses found along the meridional section in the Indian Ocean to estimate its turnover time and understand its behavior, and (3) provide spatially high-resolution FDOM data (in Raman units) that can be directly compared with data collected in other oceanic locations in the past or future.

## Materials and Methods

In this study, we analyzed FDOM data obtained from depths below 250 m in the Indian Ocean. Strictly speaking, FDOM should be called “fluorescent organic matter (FOM)” because fractional particulate organic matter (POM) might also be included in the FDOM data measured by an *in situ* fluorometer. Although POM is a minor component of organic matter at depths below 200 m in the open ocean (Catalá et al., 2015a), the term FOM is used throughout this paper accordingly.

### Vertical Section Observations

**FIGURE 1:**
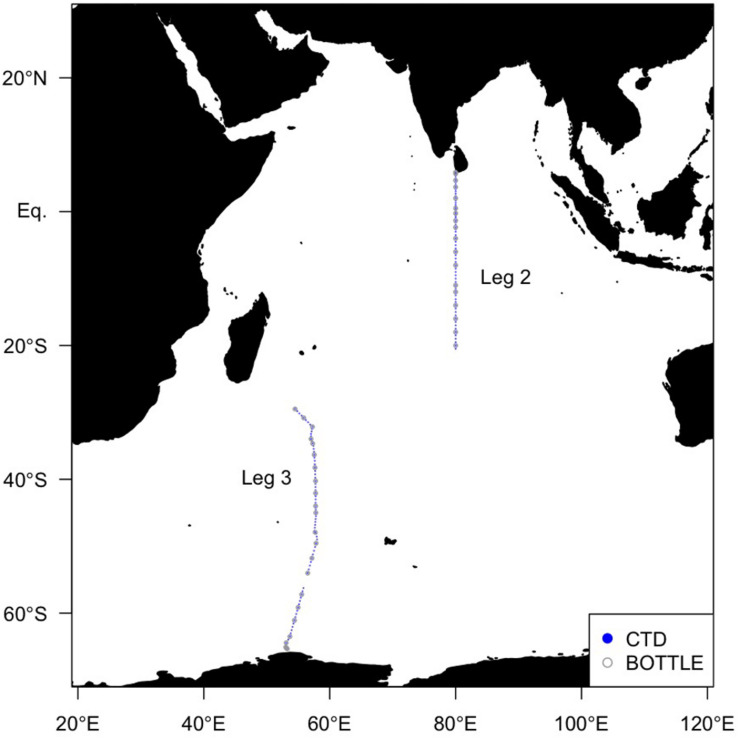
Map of sampling sites in the Indian Ocean in this study. Solid blue circles indicate sites where the FOM data were obtained using an *in situ* fluorometer, and open gray circles indicate sites where the FOM data were also measured using a shipboard benchtop fluorometer.

Global Ocean Ship-based Hydrographic Investigations Program (GO-SHIP) cruises aboard the R/V *Mirai* (MR19-04, Legs 2 and 3) were conducted along two transects with station spacing less than 67 km (Figure 1). Leg 2 covered the northern transect (December 5–27, 2019) and Leg 3 covered the southern transect (December 29, 2019–February 10, 2020). The 1 dbar interval temperature, salinity, and pressure data were obtained using the SBE9plus CTD system (Sea-Bird Scientific, United States) mounted on a 36 Niskin bottle rosette. The FOM and dissolved oxygen (DO) data were obtained using an *in situ* fluorometer (Ultraviolet fluorometer, Seapoint Sensors Inc., United States) and a DO sensor (RINKO III, JFE Advantech Co., Ltd., Japan) connected to the CTD system, respectively. The excitation and emission wavelengths of the *in situ* fluorometer were 370 nm with 12 nm FWHM and 440 nm with 40 nm FWHM, respectively (where FWHM is the full width at half maximum wave height). Hereafter, FOM measured by the *in situ* fluorometer is referred to as “FOM370/440”. This wavelength range represents that of a terrestrial humic-like fluorophore, similar to Peak C (Coble, 2007). The excitation wavelength of 370 nm is at the upper limit for humics (Chen et al., 2003). Photosynthetically Available Radiation (PAR) and turbidity data collected by a PAR sensor (PAR-Log ICSW, Satlantic LP, Canada) and a turbidity sensor (Turbidity meter, Seapoint Sensors Inc., United States) as well as nitrate and phosphate data were also used. Nitrate and phosphate data are from the Carbon Dioxide Information and Analysis Center^1^.

The DO concentrations obtained by the sensor were calibrated using concentrations measured by the Winkler titration method, as in Uchida et al. (2010). For the FOM370/440 outputs from the *in situ* fluorometer, the following two corrections were applied: (1) The differences between the outputs of the down- and up-casts that might be attributed to pressure hysteresis of the fluorometer were determined. The up-cast data were corrected to match the down-cast data as follows:

(1) $${\text{FOM}}_{c}={\text{FOM}}_{m}\times \left(1.0+c_{0}\times \left[P_{\text{lim}}-P\right]\right)\left[\text{for}P<P_{\text{lim}}\right]$$

where *P* is pressure (dbar), subscripts *c*, *m*, and *lim* indicate the corrected and measured FOM370/440 values and the threshold pressure value, respectively; and *c*_0_ is the correction coefficient. If *P* is greater than *P*_lim_, *c*_0_ was set to zero. If the maximum pressure of a given cast was smaller than *P*_lim_, *P*_lim_ was set to the maximum pressure. The *P*_lim_ and *c*_0_ pairs used for Leg 2, Leg 3 stations 70–102 and Leg 3 stations 103–153 were 2500 dbar and 1.5 × 10^–5^, 2500 dbar and 1.8 × 10^–5^, and 3000 dbar and 0.4 × 10^–5^, respectively. The down-cast data were used for the vertical sections and the up-cast data were used for comparisons with the bottle data (explained in the subsection “Temperature *D*ependence *C*orrection and *C*alibration of *R*aw *D*ata *F*rom the *in situ F*luorometer”). (2) Large positive deviations (maximum of 0.33 V) in the outputs of the *in situ* fluorometer were observed near the surface, likely due to interference from sunlight. Therefore, when the *in situ* PAR data were greater than 100 μE m^–2^ s^–1^, the near surface deviated data were replaced by the first minimum value observed below the surface in each cast. Although the FOM370/440 data might be affected by turbidity, we established no correlations between FOM370/440 and turbidity from the data obtained (*r*^2^ = 0.01), including the data from north of the Equator (*r*^2^ = 0.00), where relatively high turbidity values were found, even in the deep waters (Figure 8A). This indicates that FOM370/440 is not influenced by turbidity in this study.

### Determining the Temperature Dependence of the *in situ* Fluorometer

In the open ocean, correcting for the temperature dependence of the *in situ* fluorometer is required (Watras et al., 2011; Yamashita et al., 2015). We performed two experiments for determining the temperature dependence before the cruises. The *in situ* fluorometer was submerged in a beaker fixed in a temperature controlled water bath. Before the experiments, the beaker was washed with detergent and rinsed with Milli-Q water obtained (*R* > 18 MΩ cm^–1^) using a MilliQ IQ 7000 system (Merck) connected to an ElixUV 70 (Yamato Scientific Co., Ltd.). The temperature of the water bath was first increased to ∼30°C, then was gradually lowered to ∼1.5°C. Data were collected continuously at 1 s intervals. Using this system, we investigated the temperature dependence of the fluorometer in both Milli-Q water as a blank and in Multi-parametric Standard Seawater (MSSW) (lot Pre18) (Uchida et al., 2020), which was used to represent seawater.

### Water Sampling

In order to calibrate the FOM370/440 data collected by the *in situ* fluorometer, water samples for calibration were collected from the surface to 10 m above the bottom at each of the 41 stations (∼24 depths per cast) using 12 L Niskin bottles mounted on a rosette (Figure 1). Each sample collected in the upper 250 m was filtered using a pre-combusted Whatman GF/F glass fiber filter. Filtrations were carried out by connecting the spigot of the Niskin bottle through a silicone tube to an inline plastic filter holder. Filtrates or non-filtered seawater samples (below 250 m depth) were collected in pre-combusted glass vials with acid-washed Teflon-lined caps after triple rinsing. The samples were kept in the dark in a refrigerator at 4°C until analysis. Because silicone tubes occasionally cause the sorption of organics (Spencer and Coble, 2014; Wilde et al., 2014), we conducted a preliminary experiment. The duplicate seawater samples were collected at seven depths (from ∼200 to 5000 m) at station K2 (47°N, 160°E) from July 19 to August 10, 2018 aboard the R/V *Mirai* (MR18-04). At each depth, one sample was filtered as above, and the other was not filtered. The samples were stored frozen in the dark until analysis. The samples were then thawed, acclimated to a laboratory temperature, and measured, as described in subsection “Shipboard Measurements of FOM370/440”. The estimated standard deviation from the difference of duplicate measurements was 0.0004 (RU), which is smaller than the analytical error of 0.0005 (RU) for an *in situ* fluorometer (Table 1). Thus, the effect of the silicone tube on the FOM370/440 measurements is negligible.

**TABLE 1:** Seawater properties of the source water masses.

Source water mass abbreviation	Full water mass name	θ (°C)	*S* (psu)	*O* (μmol kg^–1^)	PV (×10^–12^ m^–1^ s^–1^)	References
WSW	Weddel Shelf Water	–1.85	34.246	314	–160.0	Johnson (2008)^1^
ALBW	Adélie Land Bottom Water	–0.55	34.678	240	–11.0	Johnson (2008)^1^
NADW	North Atlantic Deep Water	3.32	34.894	291	5.6	Johnson (2008)^1^
AAIW	Antarctic Intermediate Water	4.42	34.180	240	–132.0	You (2002)^2^
SAMW	Sub-Antarctic Mode Water	8.75	34.580	250	–34.0	Tomczak (2005)^3^
STMW	Subtropical Mode Water	16.44	35.520	214	–170.0	Tsubouchi et al. (2016)^4^
IIW	Indonesian Intermediate Water	4.83	34.606	99	–18.0	Emery and Meincke (1986)^5^; Talley and Sprintall (2005)^5^
RSOW	Red Sea Overflow Water	12.05	35.897	14	41.0	Johnson (2008)^1^
PGW	Persian Gulf Water	16.63	36.240	11	267.0	Shenoi et al. (1993)^6^
LIUW	Lower Indonesian Upper Water	10.96	34.574	103	–185.0	Emery and Meincke (1986)^5^; Talley and Sprintall (2005)^5^
UIUW	Upper Indonesian Upper Water	24.01	34.506	159	–661.0	Emery and Meincke (1986)^5^; Talley and Sprintall (2005)^5^
ICW	Indian Central Water	20.00	36.000	227	–272.0	Karstensen and Quadfasel (2002)^7^

*When the literature values for O and PV were not available, we estimated the values from the WOA18 data. The observed O and PV are shown in Figures 6G–H and I–J. ^1^O and PV were extracted from the WOA18 annual data for the locations of the specific source water masses. ^2^O was calculated by multiplying phosphate by 170 (Anderson and Sarmiento, 1994), and PV was estimated by dividing its PV value by a gravitational acceleration of 9.8 (m s^–2^). ^3^PV was extracted from the austral winter WOA18 data at the base of the winter mixed layer. Mixed layer depth was defined as the depth where a density increase from the sea surface value exceeds 0.125σ_θ_. SAMW was considered to have a PV minimum lower than 50 (×10^–12^ m^–1^ s^–1^) within the density range (26.6 < σ_θ_ < 26.9) according to Hong et al. (2020). ^4^O was extracted from the austral summer WOA18 data according to the method of Tsubouchi et al. (2016). ^5^θ, S, O, and PV were extracted from the WOA18 annual data. Referring to Emery and Meincke (1986) and Talley and Sprintall (2005), three water masses were defined within the latitudinal range (−18 to −8°S) and longitudinal range (120 to 125°E) as follows: UIUW (60 m to 31.5σ_1_ with θ higher than 17°C and salinity less than 35), LIUW (60 m to 31.5σ_1_ with θ less than 17°C and salinity less than 35), and IIW (31.5–32.2σ_1_ with salinity less than 34.7). 60 m is the maximum mixed layer depth in this region. ^6^θ, S, O, and PV were extracted from the WOA18 annual data. Referring to Shenoi et al. (1993), each value was calculated between 26.2 and 26.8 σ_θ_ within the latitudinal range (22 to 25°N) and longitudinal range (60 to 65°E). ^7^PV was extracted from the austral winter WOA18 data. PV was estimated from the linear relationship between potential temperature and PV in the same waters as in Karstensen and Quadfasel (2002).*

### Shipboard Measurements of FOM370/440

EEM fluorescence spectra were measured onboard using a benchtop fluorometer (Aqualog, Horiba Scientific, Japan) after the samples were acclimated to laboratory temperature in the dark. The measurements were conducted within two days of sampling. Emission scans from 248–829 nm were obtained at 2.33 nm intervals for sequential excitations from 240–560 nm at 5 nm intervals, with an integration time of 12 s and using the high Charge-Coupled Device (CCD) gain mode. The bandpass was fixed to 5 nm for both excitation and emission. Absorbance spectra were simultaneously collected with the benchtop fluorometer. Blank subtraction, inner filter effect correction (McKnight et al., 2001), and normalization of fluorescence intensities to Raman Units (RU) (by peak area integration of Raman scatter at an excitation wavelength of 350 nm) (Lawaetz and Stedmon, 2009) were conducted as post-measurement steps. The spectra of Milli-Q water were measured on every EEM measurement day and used for the blank subtraction and Raman normalization. The fluorescence intensities corresponding to those obtained by the *in situ* fluorometer were interpolated between excitation/emission wavelength pairs of 370/439.281 nm and 370/441.590 nm, and used to calibrate the data collected by the *in situ* fluorometer.

### Water Mass Analysis

There are many water masses that make up the Indian Ocean. We deconstructed the relative proportions of these water masses in the dark ocean (>250 m) by using optimum multi-parameter analysis (OMPA). Here, we considered the fractional contributions of the source water masses with characteristic salinities (*S*), potential temperatures (θ), DO (*O*), and potential vorticity (PV). Equations for a water sample *j* are:

(2) $$\sum_{i}x_{ij}\theta_{i}=\theta_{j},$$

(3) $$\sum_{i}x_{ij}S_{i}=S_{j},$$

(4) $$\sum_{i}x_{ij}O_{i}-\Delta O_{j}=O_{j},$$

(5) $$\sum_{i}x_{ij}{\text{PV}}_{i}={\text{PV}}_{j},$$

(6) $$\sum_{i}x_{ij}=1,$$

where *x*_ij_ is the proportion of the source water mass *i* included in sample *j*, θ*_i_*, *S*_i_, *O*_i_, and PV*_i_* are the prescribed potential temperature, salinity, DO, and PV of each source water mass, respectively, and θ*_j_*, *S*_j_, *O*_j_, and PV*_j_* are the observed temperature, salinity, DO and PV values in sample *j*, respectively. Δ*O*_j_ is the oxygen anomaly for sample *j* and is associated with organic matter respiration. PV ($\frac{f}{\rho }\partial \rho /\partial z$, where *f* is the Coriolis parameter and ρ is potential density referenced to the middle pressure over which the derivative of ρ with respect to depth (*z*) is estimated) was calculated from the CTD data, which were smoothed with a 50-dbar half-width Gaussian filter. This system is written in a matrix form:

(7) Ax=b+R,

where **A** is the source water mass type matrix, **b** is the observational data vector, **R** is the residual vector of the fit, and *x* is the vector of the unknown relative proportions of the source water masses and the oxygen anomaly. This system was solved using a non-negative least squares minimization after **A** and **b** were standardized using the prescribed θ, *S*, *O*, and PV data for the source water masses considered here. The weights of unity were assigned to θ and *S*. PV is a conservative quantity, but noisier than θ and *S* because it is derived from the vertical density derivative. Thus, a weight of 0.25 was assigned to PV. *O* is a non-conservative quantity, and a weight of 0.15 was assigned. A weight of mass conservation was set as the sum of the weights.

In this study, we confirmed the presence of 12 source water masses (Table 1 and Supplementary Figure 1). We classified the observational data into six domains by density and latitude. The waters (σ_θ_ > 27.5) along Legs 2 and 3 were classified as “bottom/deep” domain. The waters (27 < σ_θ_ < 27.5) along Legs 2 and 3 were classified as “northern intermediate” and “southern intermediate”, respectively. The waters (σ_θ_ < 27) along Leg 3 were considered to be “southern central.” The waters (σ_θ_ < 27) along Leg 2 were divided into the two domains: “northern central 1” for the waters (26.6 < σ_θ_ < 27) and “northern central 2” for the waters (σ_θ_ < 26.6). The properties for the source water masses were basically cited from the literature values in which the locations and the method of estimating the properties are clarified. When the literature values for *O* and PV were not available, the data of θ, *S*, and *O* from the World Ocean Atlas 2018 (WOA18) data were used (Garcia et al., 2018; Locarnini et al., 2018; Zweng et al., 2018). Details of our estimation of *O* and PV are detailed in Table 1. The OMPA results were considered invalid and the water mass fractions were set to zero in the following cases: 1) the total of water mass fractions is greater than 1.05 or smaller than 0.95, or 2) the squared norm of the residuals exceeds 0.25.

### Water Mass Proportions Collected During the Cruise

Using the proportion of each water mass *i* included in the sample *j* (*x*_ij_), we calculated the proportion of each water mass, *i*, in the total water samples collected during the cruises (%PRO_i_):

(8) $$\%{\text{PRO}}_{i}=\frac{\sum_{j}x_{ij}}{n},$$

where *n* is the number of samples collected.

### Water Mass Mixing-Weighted Average Pressures, FOM370/440, and AOU

The water mass mixing-weighted average pressures, FOM370/440, and AOU (hereafter, we refer to the average as the archetypal value) were calculated as in Álvarez-Salgado et al. (2013):

(9) $$C_{i}=\frac{\sum_{j}x_{ij}\times C_{j}}{\sum_{j}x_{ij}},$$

where *C*_i_ is the archetypal value of parameter *C* in the center of mass of water mass *i* during the cruises and *C*_j_ is the observed *C* value in sample *j*. *C*_i_ is affected by the conservative mixing of the value of *C* for each water mass in the source region. In addition, *C*_i_ for FOM370/440 and AOU is influenced by organic matter mineralization that occurred from the source region of each water mass to the center of mass along the two transects, i.e., on a basin scale (Álvarez-Salgado et al., 2013). We also calculated the standard deviation (SD_Ci_) of the *C*_i_ as:

(10) $${\text{SD}}_{C_{i}}=\frac{\sqrt{\sum_{j}x_{ij}\times {\left(C_{j}-C_{i}\right)}^{2}}}{\sum_{j}x_{ij}},$$

Finally, the archetypal values of parameter *C* in all the samples (<*C*_j_>) were calculated as in Catalá et al. (2015a, b):

(11) $$<C_{j}>=\sum_{j}x_{ij}\times C_{i},$$

In order to evaluate to what extent parameter *C* is influenced by water mass mixing and basin scale mineralization, the determination coefficient (*R*^2^) in a correlation between *C*_j_ and <*C*_j_> was used according to Catalá et al. (2015a, b). In addition, the residual standard deviation (*SD*_res_) for the linear regression between *C*_j_ and <*C*_j_> was calculated to understand the mean error caused by predicting the measured values of *C*_j_ with the regression (Table 2).

**TABLE 2:** Archetypal values of the pressure, AOU, and FOM with the proportion of source water mass.

Source water mass abbreviation	PRO (%)	Pressure (dbar)	AOU (μmol kg^–1^)	FOM (RU)
WSW	0.05	1572 ± 102	135 ± 1	0.0070 ± 0.0000
ALBW	60.01	3053 ± 2	152 ± 0	0.0090 ± 0.0000
NADW	6.57	2832 ± 5	155 ± 0	0.0091 ± 0.0000
AAIW	13.45	1953 ± 4	152 ± 0	0.0087 ± 0.0000
SAMW	4.68	670 ± 2	129 ± 0	0.0073 ± 0.0000
STMW	1.11	508 ± 2	37 ± 0	0.0043 ± 0.0000
IIW	3.84	947 ± 1	229 ± 0	0.0113 ± 0.0000
RSOW	8.78	1983 ± 5	178 ± 0	0.0100 ± 0.0000
PGW	0.30	449 ± 5	65 ± 1	0.0055 ± 0.0000
LIUW	0.63	363 ± 1	152 ± 1	0.0083 ± 0.0000
UIUW	0.03	281 ± 2	69 ± 3	0.0057 ± 0.0000
ICW	0.01	303 ± 3	58 ± 3	0.0054 ± 0.0000
*R*^2^ (*C*_j_ vs. <*C*_j_>)			0.53	0.34
SD_RES_			28	0.0016
Analytical error			1.1	0.0005

## Results

### Temperature Dependence Correction and Calibration of Raw Data From the *in situ* Fluorometer

**FIGURE 2:**
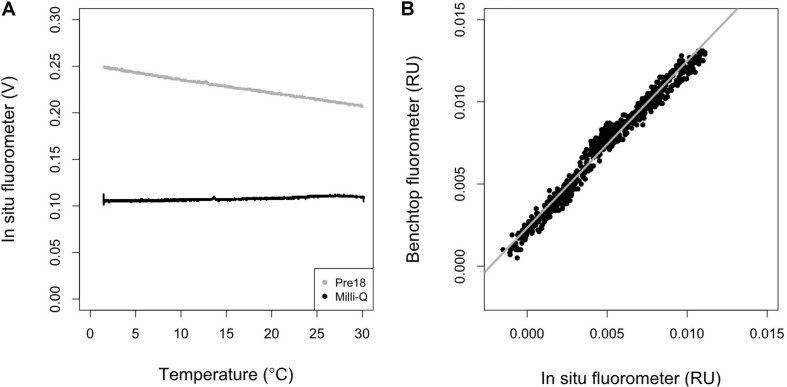
**(A)** Temperature dependence of the *in situ* fluorometer for Multiparameteric Standard Seawater, lot Pre18 (gray) and MilliQ-water (black), and **(B)** the relationship between the results obtained using a benchtop fluorometer and the outputs of the *in situ* fluorometer which were first corrected for temperature dependence and then converted to RU using the conversion factor [Eq. (13)]. In **(B)**, the gray line represents the regression line (*R*^2^ = 0.97, *n* = 769, *p* < 0.001).

The representative seawater sample (MSSW Pre18) exhibited a clear temperature dependence, while the Milli-Q water did not (Figure 2A). The response of Milli-Q water to temperature change was the same as in Watras et al. (2011). However, different from the findings of Watras et al. (2011), we measured significantly higher levels of FOM370/440 than zero in our “temperature dependence” experiment. The signals include both the blank level of FOM370/440 in the Milli-Q water and the dark count intrinsic to the fluorometer. In this study, the following temperature dependence of the *in situ* fluorometer was assumed, as in Watras et al. (2011) and Yamashita et al. (2015):

(12) $$\mathrm{FOM370}/440_{c}=\mathrm{FOM370}/440_{m/}\left(1.0+d\times \left[T-T_{r}\right]\right),$$

where *T* is temperature (°C), *T*_r_ is the reference temperature (°C), *d* is the temperature coefficient (°C^–1^), and subscripts *c* and *m* represent the corrected and measured values. A *T*_r_ of 20°C was used in this study. *d* is considered to be the ratio of the slope to the intercept in the linear regression of the signals of the *in situ* fluorometer and temperature, and is constant over a wide range of FOM370/440 levels because both the slope and intercept change proportionally with the FOM370/440 level (Watras et al., 2011). This condition holds when a null FOM370/440 level corresponds to a 0 V signal obtained by an *in situ* fluorometer. Therefore, the Milli-Q water value, including the blank level of FOM370/440 and dark count intrinsic to the fluorometer, should be subtracted from the values obtained for the Pre18 seawater measurements. Here, the average value of 0.1075 V (FL_MQ) was subtracted from the Pre18 seawater measurements. Then, Eq. (12) was fit to the Pre18 seawater measurements and *d* was estimated to be −0.013 ± 0.000 (°C^–1^). Using the *d*-value, we then corrected the temperature dependence in the data collected by the *in situ* fluorometer.

The FOM370/440 value for the Pre18 seawater (FOM_pre18) was independently measured using a benchtop fluorometer, and was estimated to be 0.0174 ± 0.0002 RU (*n* = 5). Using this value, we estimated a conversion factor, *c*, for the *in situ* fluorometer’s raw data to RU as follows:

(13) c=FOM_pre18/(FL_pre18-FL_MQ),

where FL_pre18 is the measured value (V) at 20°C by the *in situ* fluorometer for the Pre18 seawater. Here, FOM370/440_c_ was multiplied by *c* and converted to the *in situ* fluorometer data in RU. We compared the corrected data with all the data obtained using the benchtop fluorometer for the *in situ* samples and a significant positive linear correlation (*R*^2^ = 0.97, *n* = 769, *p* < 0.001) was obtained (Figure 2B). The corrected data tend to be systematically smaller values than the benchtop fluorometer data, which might be caused by the Milli-Q water containing a small amount of FOM370/440. Nevertheless, the significant linear relationship could be used to calibrate the corrected data collected during the cruises. The residual standard deviation of the linear regression between the corrected data and the benchtop fluorometer data was estimated as 0.5 mRU. This standard deviation is considered to be the analytical error of the *in situ* fluorometer in this study (Table 2). Finally, the linear relationship was used to calibrate the corrected data in RU and the calibrated data were used for the following analyses.

### Relative Proportions of Source Water Masses

**FIGURE 3:**
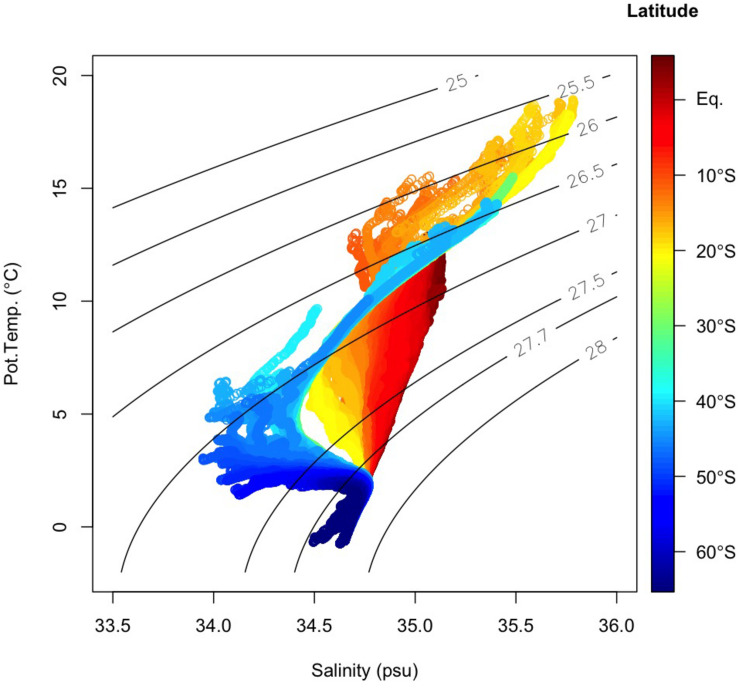
T–S (potential temperature vs. salinity) diagram below 250 m. Color represents the latitude where each sample was collected. Black lines indicate the density surfaces of σ_θ_.

In the samples collected in the dark ocean (>250 m), potential temperatures ranged from approximately −1.5 to 18°C, and salinities ranged from ∼33.9 to ∼35.8 psu (Figures 3, 6A–D and Supplementary Figure 1). As stated above, we divided the observational data into six domains: bottom/deep, southern intermediate, northern intermediate, southern central, northern central 1 and northern central 2 (Supplementary Figure 1). Twelve water masses were confirmed (Table 1 and Supplementary Table 1): Weddel Shelf Water (WSW), Adélie Land Bottom Water (ALBW), North Atlantic Deep Water (NADW), Antarctic Intermediate Water (AAIW), Sub-Antarctic Mode Water (SAMW), Subtropical Mode Water (STMW), Indonesian Intermediate Water (IIW), Red Sea Overflow Water (RSOW), Persian Gulf Water (PGW), Lower Indonesian Upper Water (LIUW), Upper Indonesian Upper Water (UIUW), and Indian Central Water (ICW). For each domain, we conducted an OMPA analysis.

**FIGURE 4:**
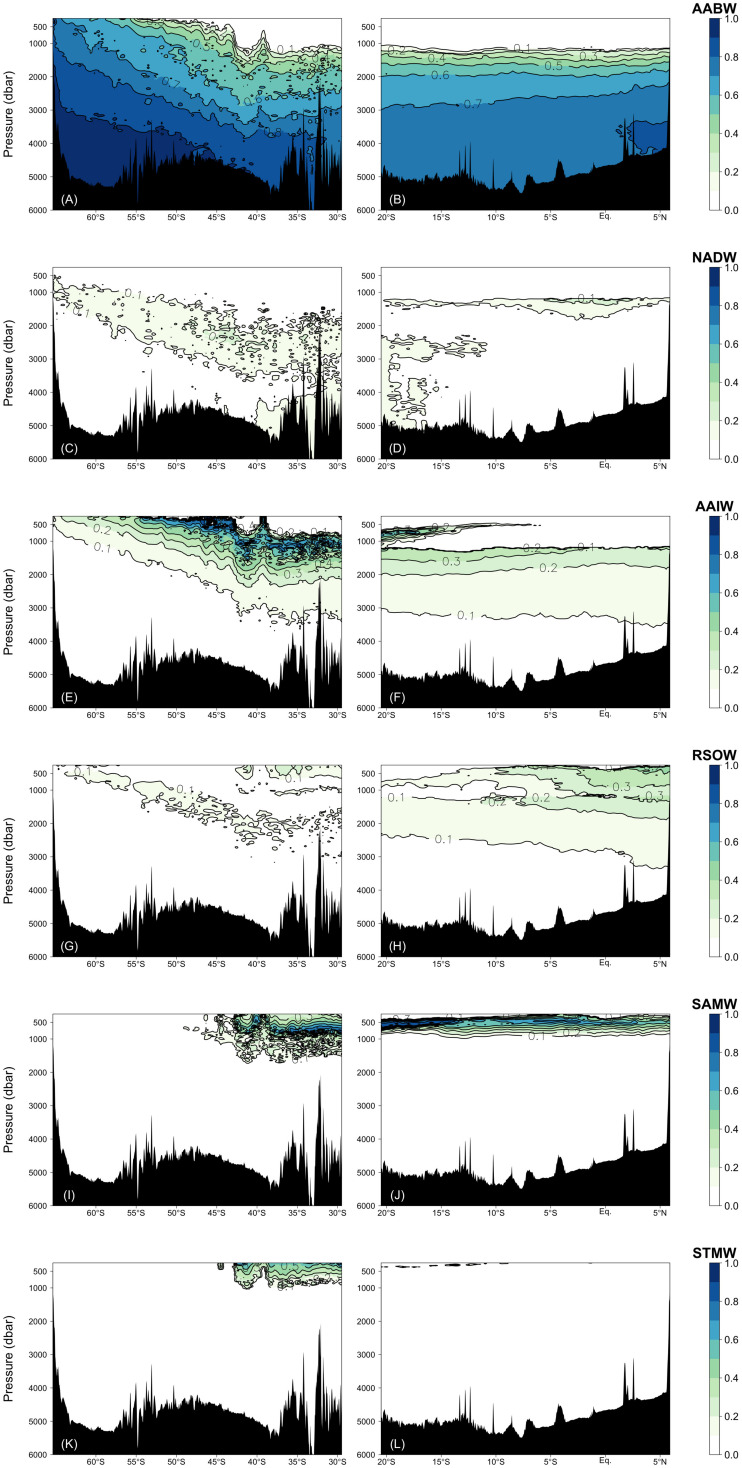
Fractions of the water masses confirmed by the water mass analyses: **(A,B)** AABW = WSW + ALBW, **(C,D)** NADW, **(E,F)** AAIW, **(G,H)** RSOW, **(I,J)** SAMW, and **(K,L)** STMW. Right and left columns represent the Leg 2 and Leg 3 transects, respectively.

The water mass that most occupied the samples along the two transects was ALBW (60.01%) (Table 2). Along both transects, Antarctic Bottom Water (AABW), which contains WSW and ALBW, played a more dominant role in filling the bottom waters compared to NADW (Figure 4). This is evident from the fact that the ALBW archetypal pressure of 3053 dbar exceeded the NADW archetypal pressure of 2832 dbar, and the occupied samples of NADW are 6.57%.

North of 40°S, the NADW fraction is approximately 0.1 and the fraction is greater than 0.2 only at about 1200 dbar near the Equator. South of 40°S, the NADW fraction exceeds 0.2 within the latitudinal range of −50 to −40°S, and the core tilts toward the south along the isopycnals (Figures 4 and 6E). The distributions of AABW and NADW are similar to the previous results (Johnson, 2008).

From the south, AAIW, SAMW, and STMW mainly ventilate the waters above the bottom/deep water masses and spread northward. The occupied samples of AAIW, SAMW, and STMW were 13.45, 4.68, and 1.11%, respectively. The archetypal pressure of AAIW was 1953 dbar. The pressures of SAMW and STMW were 670 and 508 dbar, respectively, which are shallower than that of AAIW.

**FIGURE 5:**
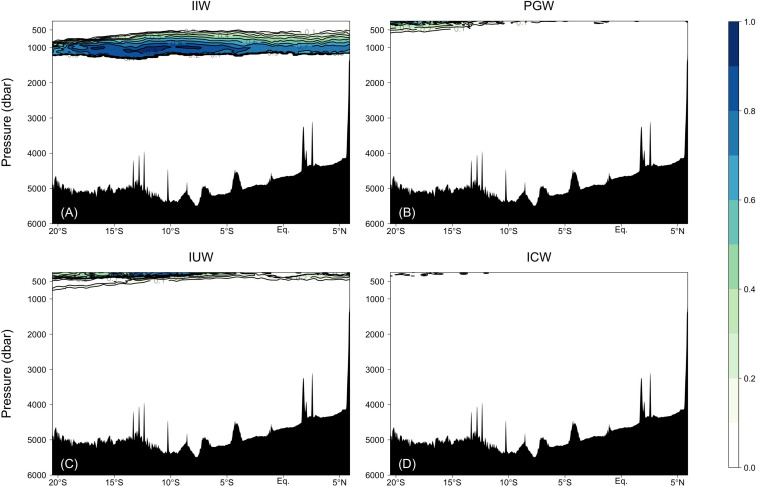
Fractions of the water masses confirmed by the water mass analyses: **(A)** IIW, **(B)** PGW, **(C)** IUW = UIUW + LIUW, and **(D)** ICW.

In the intermediate waters of the Leg 2 transect, RSOW and IIW were the dominant water masses. RSOW spread from the north to the south, and the proportion of occupied samples was 8.78%. The archetypal pressure of RSOW was 1983 dbar. IIW spreads from the Indonesian seas westward (Talley and Sprintall, 2005) and was located between ∼700 and 1200 dbar. The core was centered at ∼5–18°S and at ∼1000 dbar and the proportion of occupied samples was 3.84% (Table 2 and Figure 5).

PGW, IUW and ICW were minor water masses in this study. PGW, which spreads from the north, was observed above 500 dbar south of the Equator. IUW was also observed above 500 dbar over a latitudinal range of Leg 2 transect, with a volume (LIUW + UIUW) of 0.66%. This water mass spreads from the Indonesian seas as the Indonesian throughflow (Talley and Sprintall, 2005). ICW with a proportion of occupied samples of 0.01% was located from ∼12 to 20°S and above 400 dbar. Vertical sections of fractions of WSW and PGW along the Leg 3 transect, and UIUW and LIUW along the Leg 2 transect are presented in the Supplementary Figure 2.

### Distributions and Relationships Between FOM370/440 and AOU

**FIGURE 6:**
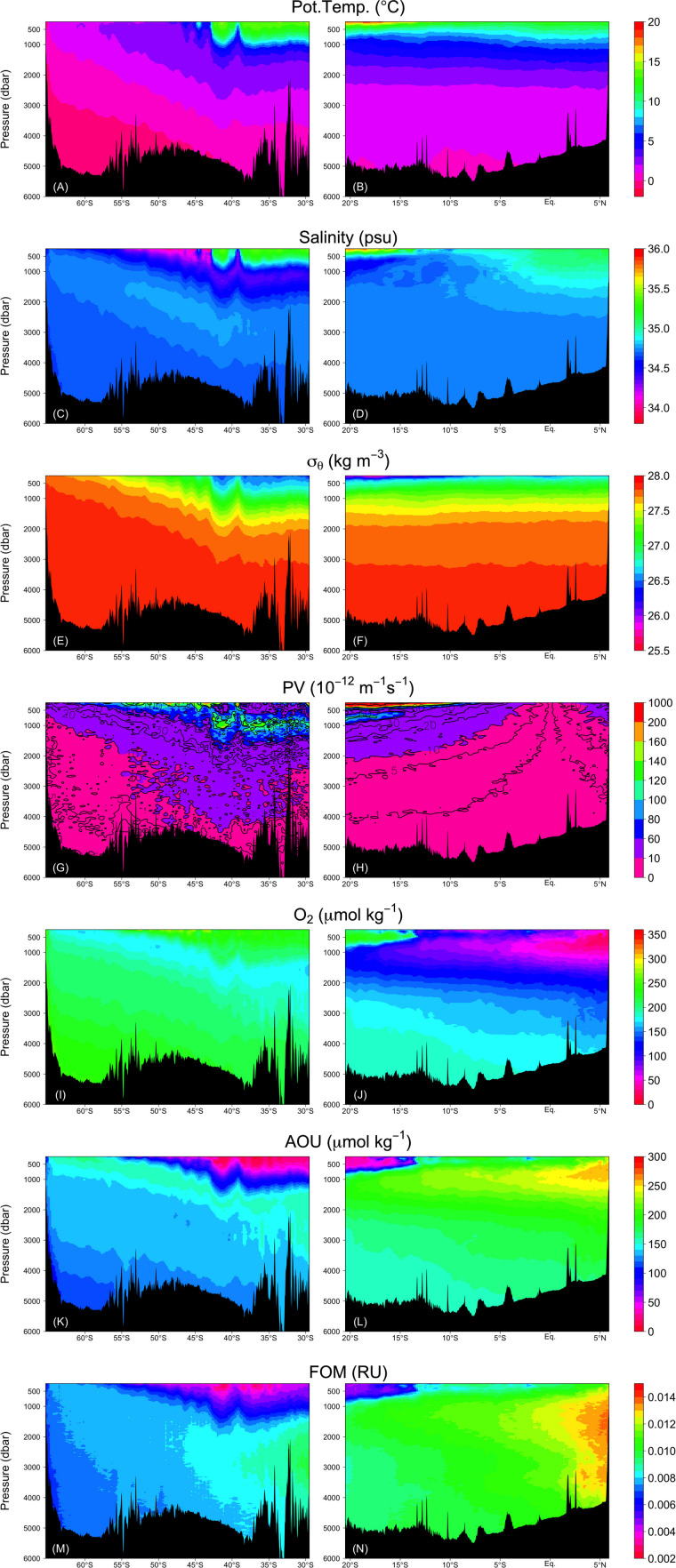
Vertical sections of **(A,B)** potential temperature, **(C,D)** salinity, **(E,F)** σ_θ_, **(G,H)** absolute value of PV, **(I,J)** dissolved oxygen, **(K,L)** AOU, and **(M,N)** FOM. Right and left columns represent the Leg 2 and Leg 3 transects, respectively.

FOM370/440 levels are generally low in the upper ocean where photodegradation is the main sink process (e.g., Chen and Jeffrey, 1992) and increases with depth (Figure 6M,N). FOM370/440 levels ranged from approximately 0.002 to 0.014 RU. At the abyssal waters (>27.75 σ_θ_), FOM370/440 levels increased from south to north. At the northern end of the Leg 2 transect (north of the Equator), the FOM370/440 levels between 1200 dbar (>∼27.5 σ_θ_) and the seafloor are comparable to those between 500 and 1200 dbar (Figures 6E–F and M–N).

AOU varied between 0 and 300 μmol kg^–1^ and had a similar spatial distribution as FOM370/440, except in the waters below 1200 dbar north of the Equator (Figures 6K,L). In contrast to FOM370/440, AOU levels between 1200 dbar and the seafloor at the northern end of the Leg 2 transect (north of the Equator) were clearly lower than those between 500 to 1200 dbar. Based on our analyses, 53 and 34% of the variability in AOU and FOM370/440 were explained by the archetypal AOU and FOM370/440, respectively (Table 2), which means that approximately half and approximately 30% of the AOU and FOM370/440 variability were determined by water mass mixing and basin scale organic matter mineralization.

**FIGURE 7:**
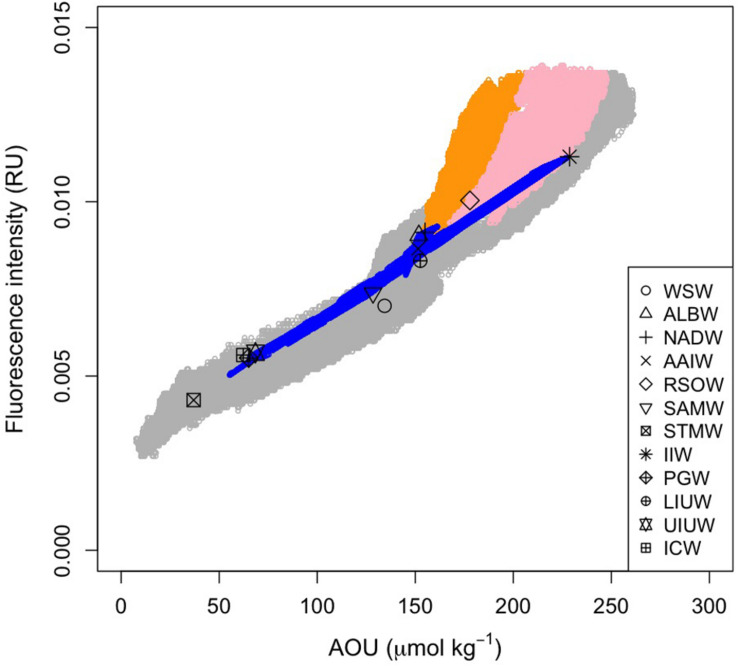
Relationship between FOM and AOU. Open gray and blue circles indicate the measured and archetypal values, respectively. Orange and pink dots indicate the measured data in the density ranges of σ_θ_ > 27.75 and 27.75 > σ_θ_ > 27.5 along the Leg 2 transect, respectively. Symbols are the archetypal values for each of the water masses.

The maximum archetypal AOU and FOM370/440 values were observed in the northern intermediate water masses: IIW (229 μmol kg^–1^, 0.0113 RU) and RSOW (178 μmol kg^–1^, 0.0100 RU) (Table 2 and Figure 7). The minimum values were observed in the upper water masses: STMW (37 μmol kg^–1^, 0.0043 RU), ICW (58 μmol kg^–1^, 0.0054 RU), PGW (65 μmol kg^–1^, 0.0055 RU), and UIUW (69 μmol kg^–1^, 0.0057 RU). Compared to the four upper water masses, the other upper water masses (LIUW) have relatively high AOU and FOM370/440 values. As for the abyssal/deep water masses, WSW, ALBW, and NADW had archetypal AOU and FOM370/440 values between the extremes mentioned above. AAIW and SAMW had similar AOU and FOM370/440 values to those of the abyssal/deep water masses.

Here, we used AOU as a proxy for microbial respiration because approximately 50% of the AOU variability was determined by water mass mixing and basin scale organic matter mineralization processes. As we anticipated from the vertical sections of AOU and FOM370/440, the archetypal AOU had a significant positive correlation with the archetypal FOM370/440 (FOM_i_ = (3.7 ± 0.2) × 10^−5^ × AOU_i_ + 0.0030 (± 0.0003), *r*^2^ = 0.96, *p* < 0.001, *n* = 12, Figure 7).

### Basin Scale Turnover Time of FOM

According to the method of Catalá et al. (2015a, b), we estimated the net production rate of FOM370/440. The net production rate (NP_FOM_) can be estimated as follows:

(14) $${\text{NP}}_{\text{FOM}}=\frac{\partial \text{FOM}}{\partial \text{AOU}}\times \text{RESP},$$

where $\frac{\partial \mathrm{FOM}}{\partial \mathrm{AOU}}$ is the rate of change in FOM370/440 with respect to AOU, and RESP represents the respiration rate in the dark ocean. We used a slope of (3.7 ± 0.2) × 10^–5^ in the linear regression between the archetypal FOM370/440 and AOU as $\frac{\partial \mathrm{FOM}}{\partial \mathrm{AOU}}$ and adopted the respiration rate that was calculated as follows: the total oceanic respiration rate (827 T mol year^–1^) below 200 m (Andersson et al., 2004) divided by the mass of the ocean below 200 m depth (1.38 × 10^21^ kg; Pilson, 1998) produces an estimate of the respiration rate (0.6 μmol kg^–1^ year^–1^) according to Catalá et al. (2015b). Using this estimated respiration rate, NP_FOM_ was estimated as 2.2 ± 0.1 × 10^–5^ RU year^–1^. The FOM370/440 turnover time (*τ*) in the Indian Ocean was then calculated using $\tau =\frac{\sum_{i}{\text{PRO}}_{i}\times \text{FOM}370/440_{i}}{{\text{NP}}_{\text{FOM}}}$, which yielded an estimated turnover time of 410 ± 19 year.

## Discussion

### Temperature Coefficient in the Temperature Dependence Curve of the *in situ* Fluorometer

The *d* of −0.013 estimated in this study is similar to the values (−0.015 to −0.016) obtained for the same type of *in situ* fluorometer (Ultraviolet Fluorometer from SeaPoint Sensors, Inc.) by Watras et al. (2011). Their estimates were obtained by conducting a similar experiment to this one on samples obtained from wetland-dominated lakes. Because the water in the lakes has completely different organic matter matrices and concentrations from water in the open ocean, the similarity in *d*-values indicates that *d* is likely related to the characteristic temperature dependence, i.e., the thermal quenching properties (e.g., Baker, 2005), of specific organic matter that was determined at fixed excitation and emission wavelengths of 370 and 440 nm, respectively. This is in line with the inference by Yamashita et al. (2015).

Watras et al. (2011) also determined the temperature dependence of another type of *in situ* fluorometer (C3 Submersible Fluorometer, TurnerDesigns, Inc., United States), whose excitation and emission wavelengths are 340 and 440 nm, respectively. They estimated *d*-values of −0.007 to −0.009 for the water samples obtained in the same wetland-dominated lakes. In addition, Yamashita et al. (2015) estimated the *d*-values for open ocean water as −0.005 to −0.006 using an *in situ* fluorometer [ECO-FL(RT)D, WET Labs, United States] with excitation and emission wavelengths of 370 and 460 nm, respectively. Although it is possible that the differences in *d*-values between the different types of *in situ* fluorometers might be instrument-specific, there is also the possibility that the differences are related to how the *d*-value is estimated. In this study, Milli-Q water had positive values, as stated above. If we estimate the *d*-value for the Pre18 seawater measurements without subtracting the Milli-Q water value, we obtain a *d*-value of −0.007, which is close to the results obtained by Yamashita et al. (2015). However, when applying this value to correct the raw data collected by the *in situ* fluorometer in this study, the temperature dependence correction did not work well. Ideally, at a null FOM370/440 fluorescence intensity, the slope and intercept of the linear regression between temperature and FOM370/440 fluorescence should approach zero. However, when the Milli-Q water value is not subtracted from the Pre18 seawater values, the slope and intercept at the FOM370/440 fluorescence intensity of Milli-Q water (i.e., ∼0.1 V) do not approach zero (Figure 2A). As a result, an incorrect *d* is estimated. Therefore, in addition to measuring a water sample with a given concentration of FOM, as suggested by Watras et al. (2011), null FOM water (such as Milli-Q water) should also be measured. The FOM value includes a blank level of FOM and dark counts that are intrinsic to an *in situ* fluorometer, and must be subtracted from the values of a water sample before estimating its *d-*value. Furthermore, the slight differences in excitation and emission wavelengths between the fluorometers might also be partly responsible for the differences in estimated *d-*values. This is because the humic-like fluorophores determined by each pair of excitation and emission wavelengths may have slightly different thermal quenching properties.

### FOM370/440 Turnover in the Indian Ocean and on a Global Scale

In this study, we observed a significant positive correlation between the archetypal AOU and FOM370/440, similar to previous studies (e.g., Catalá et al., 2015b), which indicates that FOM370/440 is produced in the process of microbial respiration in the deep waters (>250 m depth) of the Indian Ocean. The turnover time of FOM370/440 in the Indian Ocean measured by the *in situ* fluorometer was 410 ± 19 year. The FOM determined from excitation and emission wavelengths of 370 and 440 nm, respectively, is traditionally defined as terrestrial (Coble, 2007). Thus, we can compare the estimated turnover time determined in this study with that of the humic-like FOM component 1 in Catalá et al. (2015b), which was estimated for samples not filtered using a combination of EEM and PARAFAC analyses. Although the sampling in their study was carried out only in the subtropical region of the Indian Ocean (transect centered at ∼40°S) and the meridional sampling coverage in the basin was low, the estimated turnover time of the humic-like FOM component 1 on a global scale was 435 ± 41 years. Comparing our estimate with that of Catalá et al. (2015b), we can conclude that the production rate of this type of humic-like fluorophore per unit oxygen consumption is, on average, similar on a global scale. That is because the respiration rate used in this study is the same as that in Catalá et al. (2015b). The consistency in humic-like FOM turnover time between the two studies also indicates that *in situ* fluorometer measurements are useful when appropriate corrections and calibrations are applied to the raw data. In addition, the estimate in this study (3.7 × 10^–5^) is similar to the other estimates of the production rate of this type of humic-like fluorophore per unit oxygen consumption for filtered samples, 3.493 × 10^–5^ on a global scale (Jørgensen et al., 2011) and 3.9 × 10^–5^ in the North Pacific Deep Water (Tanaka et al., 2014). We must carefully compare the rates because the former two studies used unfiltered seawater samples and the latter two studies used filtered ones. FDOM characteristics through various pore size of filters are different from each other (Baker et al., 2007; Xu and Guo, 2017; Yang et al., 2020). In addition, the latter two studies did not consider the effect of water mass mixing on the results of AOU and FDOM. However, even though the microbial community in the dark ocean differs between basins, and processing methods also differ (i.e., filtered or not filtered), the production rate per unit oxygen consumption in deep waters appears to be similar on a global scale. The FOM370/440 turnover time (∼400–470 year) is of the same order of magnitude as the circulation age of 716 year in the Indian Ocean (Matsumoto, 2007) and dissolved organic carbon (DOC) aging of ∼200–400 year along the meridional circulation of the Indian Ocean (DOC aging is estimated to be the difference in the ^14^C age of DOC between Circumpolar Deep Water (CDW) and Indian Deep Water (IDW) (Bercovici et al., 2018). This indicates that this type of humic-like fluorophore is refractory and plays an important role in sequestering reduced carbon in the dark ocean.

### Deviation From the Relationship Between Archetypal AOU and FOM370/440

The relationships between AOU and FOM370/440 in the waters (σ_θ_ > 27.75 and 27.5 < σ_θ_ < 27.75) along the Leg 2 transect (orange and pink dots in Figure 7, respectively) differ from the basin scale relationship. The abyssal waters (σ_θ_ > 27.75) are occupied by the AABW (Figures 4, 7) that flow northward from the Southern Ocean. In the course of this northward flow, FOM370/440 production is enhanced, especially along the Leg 2 transect (orange dots in Figure 7). The abyssal waters overturn in the northern Indian Ocean and entrain local intermediate waters (Talley, 2013). The altered abyssal waters, called the IDW, return toward the Southern Ocean (Talley, 2013) in the deep/intermediate waters (27.5 < σ_θ_ < 27.75) (Xue et al., 2015), which is mainly occupied by AABW, RSOW, and AAIW (Figure 4). In the course of the southward flow, FOM370/440 and AOU gradually decrease and return to the basin scale relationship (pink dots in Figure 7). Note here that we did not consider CDW due to the following reasons: 1) CDW is derived from a mixture of NADW and AABW (Talley, 2013), and 2) CDW is not from dense waters from a marginal sea or ventilation.

**FIGURE 8:**
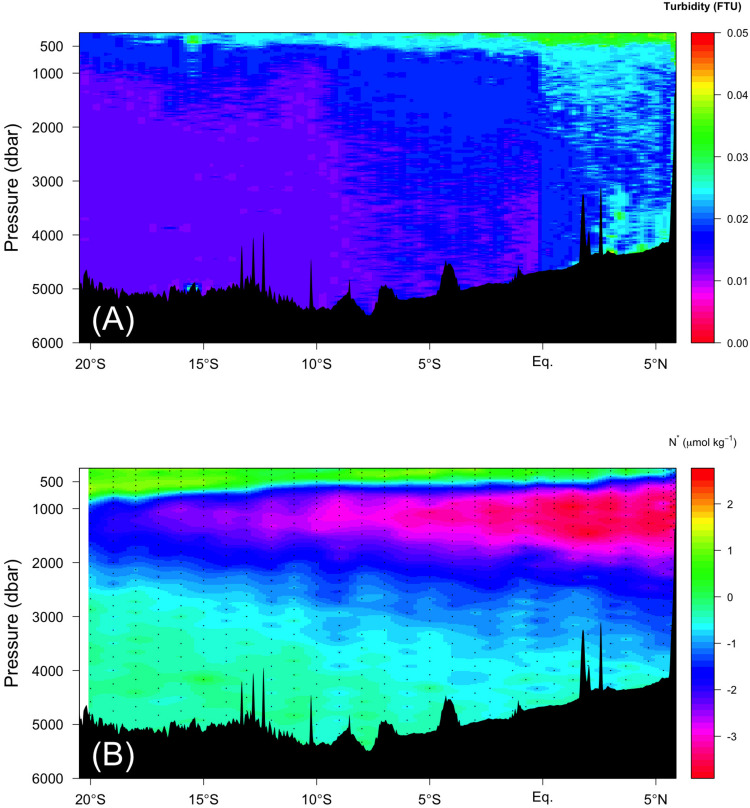
Vertical section of **(A)** turbidity (FTU) and **(B)** N* along the Leg 2 transect.

In the abyssal waters (σ_θ_ > 27.75), some mechanisms to enhance FOM370/440 production are needed. We propose the following two mechanisms: (1) FOM370/440 is supplied by hydrothermal vents, and (2) additional vertical transport of less labile POM at the northern end of the Indian Ocean, which is located below low oxygen waters (<20 μmol kg^–1^). For the first mechanism, several hydrothermal vents have been reported along the Central Indian Ridge and the Southwest Indian Ridge in the Indian Ocean (e.g., Kawagucci et al., 2008; Tao et al., 2012). Recently, Nishioka et al. (2013) observed that high dissolved Fe concentrations in the abyssal waters spread up to ∼2000 m depth from the bottom and over 3000 km laterally, at latitudes of ∼5°N–30°S along ∼70°E. They considered that the high dissolved Fe concentrations were derived from hydrothermal vent, while the extensive vertical and lateral spreading of high dissolved Fe concentrations was ascribed to dissolved Fe bound to organic ligands and/or Fe sulfide-bearing nanoparticles. If dissolved Fe bound to organic ligands causes extensive vertical and lateral transport of dissolved Fe in the abyssal Indian Ocean, there is a possibility that FOM370/440 could also be affected by the hydrothermal vents, because humic-like FDOM is likely a major Fe-binding organic ligand in the dark ocean (Laglera and van den Berg, 2009; Yamashita et al., 2020). A few studies indicate that hydrothermal systems act as a sink of DOM (Hawkes et al., 2016; Yang et al., 2017), while some studies indicate that the systems behave as a source of DOM (Yang et al., 2012; Sarma et al., 2018). In this study, the regions were not found where both turbidity and FOM370/440 levels were high along the Leg 2 transect (Figures 6L, 8A). In addition, humic acids are weakly bound to Fe and are considered to be important as Fe ligands on a short time scale (Croot and Heller, 2012) in ocean surface waters. That holds for the intermediate waters in the Pacific (Yamashita et al., 2020), while in the intermediate waters of the Atlantic, the relationship between humic acids and iron solubility are not so strong (Heller et al., 2013). Thus, based on the evidence at hand, the first mechanism might be challenging to explain the deviation.

For the second mechanism, fluxes of sinking POM passing through the oxygen-minimum zone (OMZ) are less attenuated compared to those through the oxic zone (Van Mooy et al., 2002), indicating that POM passing through the OMZ is difficult for microbes to decompose. Thus, in the northern Indian Ocean along the Leg 2 transect, POM can be transported more efficiently through the OMZ to abyssal waters compared to elsewhere along the Leg 2 and 3 transects. Even in the abyssal waters where excess FOM370/440 relative to the observed AOU was observed (Figures 6L,N and orange dots in Figure 7), a high turbidity signal was observed north of the Equator (Figure 8A). According to Van Mooy et al. (2002), microbes preferentially use nitrogen-rich amino acids in POM under OMZ-like conditions via denitrification, indicating that more labile constituents of POM are decomposed in these conditions in spite of the bulk organic matter decomposition rate being lower and more POM being transported up to the abyssal waters. Recently, Jørgensen et al. (2014) reported that the production of humic-like FDOM relative to oxygen consumption was enhanced for less labile organic substrates in long term (>1 year) incubation experiments. This mechanism may occur at the northern end of the Leg 2 transect. Although there is no direct evidence that denitrification occurs north of the Equator, there is a region where the DO concentration is less than 20 μmol kg^–1^ (Figures 6I,J), below which anaerobic microbial processes start (Rixen et al., 2020, in review), and a nitrate deficiency based on N^∗^ (nitrate – 16^∗^phosphate +2.9) is observed (Gruber and Sarmiento, 1997; Figure 8B). In the eastern Pacific, where an OMZ exists, similar decoupling of FOM from AOU was found by Catalá et al. (2015b). Thus, this mechanism deserves further examination.

## Conclusion

We obtained spatially high-resolution FOM370/440 data by using an *in situ* fluorometer in the Indian Ocean. A temperature dependence correction for the fluorometer and data calibration using the data from manually measured samples were applied. In the temperature dependence correction, measuring null FOM370/440 water, such as Milli-Q water, and subtracting the value from the value of a sample with a given FOM370/440 concentration is important for obtaining the exact temperature coefficient for the linear regression between FOM370/440 fluorescence intensity and temperature.

From the relative proportions of the water masses estimated by the OMPA analyses, we estimated the archetypal FOM370/440 and AOU values for each water mass that was considered in the analyses. We found a linear relationship between the archetypal FOM370/440 and AOU values, indicating that FOM370/440 is produced in the process of microbial respiration in the dark ocean (>250 m). We multiplied the change in FOM370/440 relative to AOU in the relationship by the microbial respiration rate in the dark ocean to estimate the turnover time of FOM370/440 in the Indian Ocean, which was of the same order of magnitude as the circulation age in the Indian Ocean. Our estimate is similar to previous results, indicating that humic-like FOM370/440 measured using an *in situ* fluorometer is a sink of reduced carbon in the dark ocean and that our correction and calibration of the *in situ* fluorometer data were successful. Applying the method for an *in situ* fluorometer attached to a CTD system and an autonomous profiling Bio-Argo float contributes to obtaining more data of humic-like FOM in the open ocean which can be directly compared with each other.

A local deviation from the archetypal FOM370/440 vs. AOU relationship was observed in the abyssal waters of the northern Indian Ocean. This local deviation is hypothesized to be affected by the FOM370/440 supply from hydrothermal vents and the effect of sinking POM altered by denitrification passing through the OMZ on the abyssal FOM370/440 production relative to oxygen consumption. Although the former process is unlikely based on the evidence at hand, these candidate processes to explain the deviation could be proposed using the high-resolution spatial FOM370/440 data obtained by an *in situ* fluorometer. Proper corrections for the temperature dependence of an *in situ* fluorometer and appropriate calibration of the corrected data make an *in situ* fluorometer useful for studying DOM in the ocean.

## Data Availability Statement

The data presented in this study are available from the corresponding author upon reasonable request.

## Author Contributions

MS and HU measured FOM using the benchtop and *in situ* fluorometers, respectively. MS performed the analyses for the results and prepared the manuscript based on advice from HU, TY, KA, and AM. All authors contributed to the design of the study.

## Conflict of Interest

The authors declare that the research was conducted in the absence of any commercial or financial relationships that could be construed as a potential conflict of interest.

## Footnotes

1 http://cdiac.ornl.gov
