# Supplementary material for: Determining the Distribution of Fluorescent Organic Matter in the Indian Ocean Using in situ Fluorometry
Source: Front Microbiol. 2020 Dec 23;11:589262. doi: 10.3389/fmicb.2020.589262 (PMC7785776; doi:10.3389/fmicb.2020.589262)
Supplement: Supplementary file 1 [file Image_1.pdf]

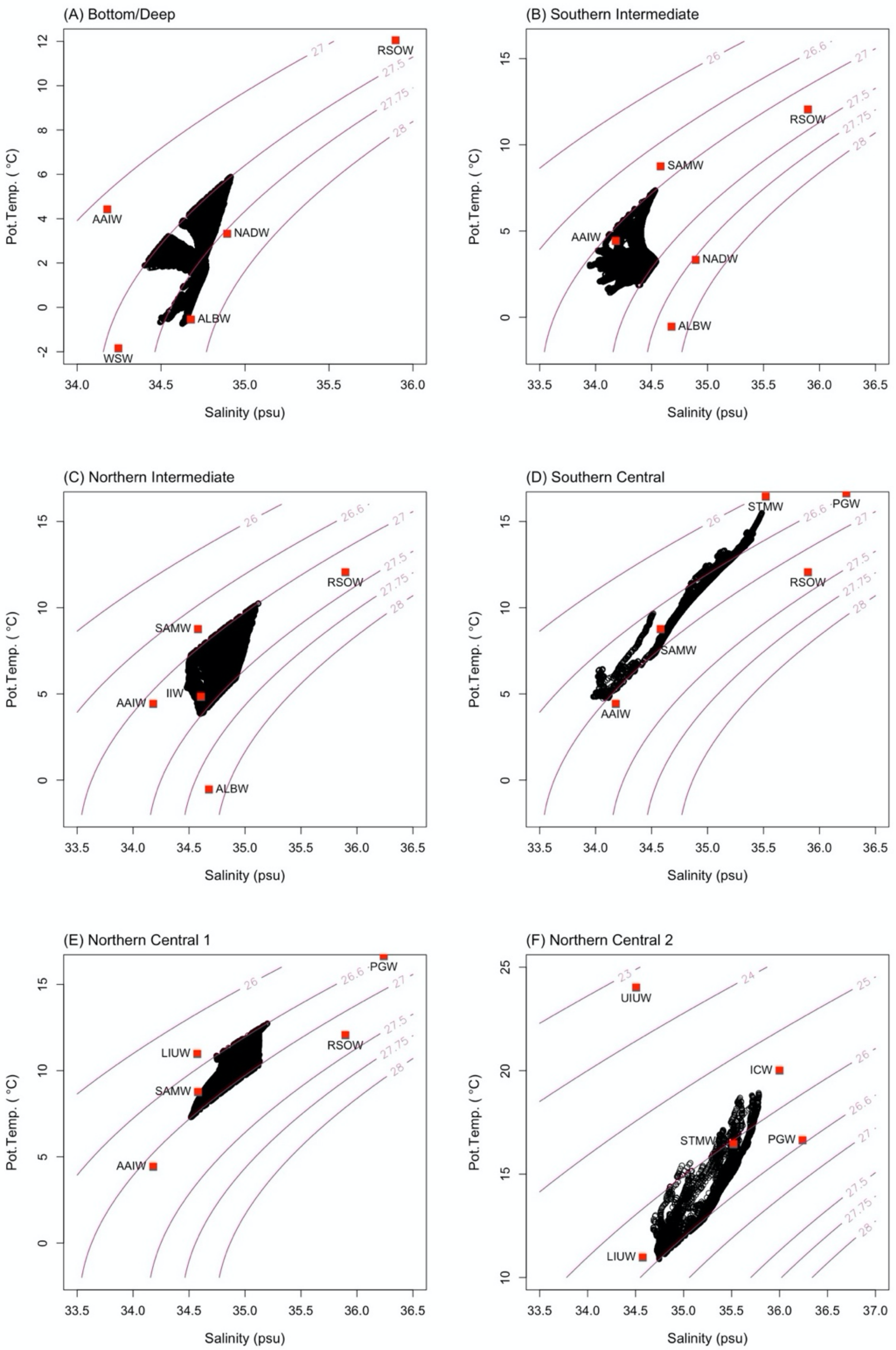

Supplementary Figure 1

T-S (potential temperature versus salinity) diagrams for the six domains used in the water mass analysis. Contours in magenta indicate the density surfaces of σ_θ. The values of potential temperature and salinity values for each water mass are shown in Table 1.
