# Supplementary material for: Determining the Distribution of Fluorescent Organic Matter in the Indian Ocean Using in situ Fluorometry
Source: Front Microbiol. 2020 Dec 23;11:589262. doi: 10.3389/fmicb.2020.589262 (PMC7785776; doi:10.3389/fmicb.2020.589262)
Supplement: Supplementary file 2 [file Image_2.pdf]

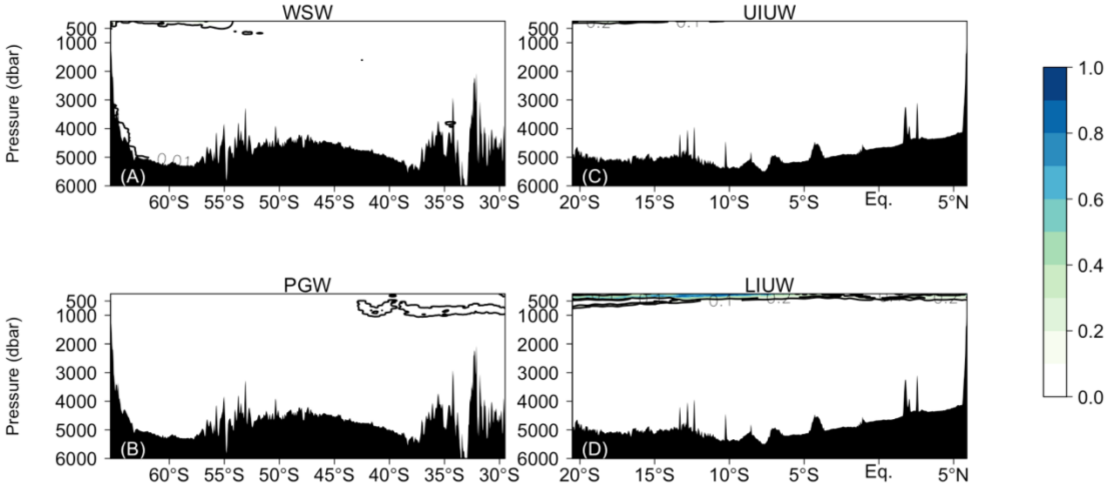


Supplementary Figure 2

Vertical sections of fractions of (A) WSW and (B) PGW along the Leg 3 transect, and (C) UIUW and (D) LIUW along the Leg 2 transect. In (A) and (B), a fraction of 0.01 is shown in a black contour, whereas in (C) and (D), fractions of 0.1 and 0.2 are shown in a black contour.
