# Supplementary material for: Determining the Distribution of Fluorescent Organic Matter in the Indian Ocean Using in situ Fluorometry
Source: Front Microbiol. 2020 Dec 23;11:589262. doi: 10.3389/fmicb.2020.589262 (PMC7785776; doi:10.3389/fmicb.2020.589262)
Supplement: Supplementary file 3 [file Table_1.pdf]

Supplementary Table 1 Descriptions of the source water masses

Source water mass	Characteristics	Reference
WSW	a near-surface contributor to AABW* found in the Weddel Sea	Johnson (2008)
ALBW	a variety of AABW* formed off Adelie Land coast of East Antarctica	Johnson (2008)
NADW	deep waters formed in the North Atlantic	Johnson (2008)
AAIW	circumpolar intermediate waters formed in the southeast South Pacific	You (2002)
SAMW	Intermediate waters formed by deep convection in late austral winter, north of the Antarctic Circumpolar Current in the southeastern part of the Indian Ocean	Tomczak (2005)
STMW	Intermediate waters formed in the western part of the Indian Ocean subtropical gyre	Tsubouchi et al. (2016)
IIW	deep core of Indonesian Throughflow Water formed in the Indonesian passages	Talley and Sprintall (2005)
RSOW	Intermediate waters introduced from the Red Sea to the northwestern part of the Indian Ocean	Johnson (2008)
PGW	Intermediate waters introduced from the Persian Gulf spill into the Gulf of Oman	Shenoi et al. (1993)
LIUW	Lower section of the shallow core of Indonesian Throughflow Water formed in the Indonesian passages	Talley and Sprintall (2005)
UIUW	Upper section of the shallow core of Indonesian Throughflow Water formed in the Indonesian passages	Talley and Sprintall (2005)
ICW	Intermediate waters formed by subduction in the latitudinal range from ~20°S to ~40°S of the Indian Ocean	Karstensen and Quadfasel (2002)

* Bottom waters formed around Antarctica (with the several source regions)
